# The American Cyclopædia of Practical Medicine and Surgery; a Digest of Medical Literature

**Published:** 1836-01

**Authors:** 


### Art. VI.

*The American Cyclopædia of Practical Medicine and Surgery; a Digest of Medical Literature.* Edited by Isaac Hays, m.d. Parts I. to VII.—*Philadelphia:* July 1833 to April 1835. 8vo. Price of each Part, fifty cents, or half a dollar.

The work before us is one of those collections which form a distinguishing feature of the activity and energy characteristic of the present era. The want of comprehensive digests of our knowledge seems to have been simultaneously felt in several of the more enlightened states of the civilized world, and accordingly Encyclopædias and Dictionaries, of various extent and merit, have been multiplied of late years in almost every country which possesses any pretensions to literary or scientific acquirements. In that department which comes more immediately within our own province, many excellent works of this description have appeared within a very short period of time; some notice of which will be found in another part of the Number.

The American Cyclopædia possesses, in common with some of the foreign works alluded to, with the Cyclopædia of Practical Medicine, lately completed, and with that of Anatomy and Physiology, now in course of publication in this country, the advantages of being written by several individuals. This plan, though necessarily liable to some objections,—such as a want of unity of thought and composition, and in some instances an undue relation in the comparative length to which the several essays are extended, —permits the assigning of the subjects treated of to those authors who have made them respectively the objects of especial study. Medicine (although the manner in which Dr. Copland's great undertaking is executed would seem almost to afford an exception to the remark,) is too extensive a science to be fully entered into in all its branches by one individual, however gifted; and here therefore, as elsewhere, the advantages resulting from the division of labour become too manifest to require pointing out. It is upon this plan, then, of the division of labour amongst several contributors, and the allotting to each that department and those subjects with which he is, from previous study, locality, or acquired advantages, most conversant, that the editor of the American Cyclopædia has proceeded in the execution of his arduous task. The work embraces within its range the whole extent of medical science,—anatomy and physiology; pathology, surgical and medical; obstetrics; therapeutics; the *materia medendi*, and pharmacy, with so much of the kindred sciences of natural history and chemistry as are necessarily connected with them; forensic medicine, and a tolerably full, though by no means complete, exposition of the scientific and technical terms used by medical authors.

The anatomical articles hitherto published are chiefly from the pen of Dr. Geddings, professor of anatomy and physiology in the University of Maryland, and evince much research and judgment in their compilation. We select the observations upon the Adipose Tissue, as affording a good insight into the method pursued by this author, and a favorable example of the manner in which he discusses the subjects allotted to him.

After distinguishing this elementary texture into the general adipose tissue, or that which occupies the superficies of the body and the surface and interstices of the organs, and the medullary, or that which is disposed within the cavities of the bones, he proceeds to consider the general adipose tissue, in relation to its quantity and proportion to the other parts of the animal economy; its distribution on the superficies, or in the interior; its progressive development; the causes influencing its increase or diminution, at different periods of life, and under varying circumstances of health or disease; and its variations in form and density, whether in different parts of the body, or at different periods of growth, maturity, and decay. The organization of the adipose tissue is then entered into at some length, and the opinions of various authors upon this curious subject brought forward and discussed. The form and size of its granulations, and the question of its constituting a distinct tissue, are then considered; and these are followed by a short notice of Chevreul's researches into its proximate elementary composition, and of M. Raspail's objections to the views of this experimenter. A brief allusion to the opinions entertained with respect to the mode of its formation, and a summary of the probable uses which the adipose tissue is intended to perform in the animal economy, conclude this part of the subject. The medullary adipose tissue is then in like manner considered; and, in a second article, the pathological anatomy, under the heads of Preternatural Development, or Hypertrophy, Preternatural Deficiency, or Atrophy, and Alterations of Texture.

The organization of the adipose tissue is an enquiry of much interest, and the question of its constituting a formation distinct from the general reticular or cellular texture, one which, notwithstanding the researches of William Hunter, Beclard, &c., on the one side, and Haller, Bichat, Cloquet, Raspail, &c., on the other, cannot even yet be considered as determined. "The adipose tissue," observes Dr. Geddings, "is of a whitish yellow colour, and, though variable in its texture in different situations, it is nevertheless everywhere composed of small, rounded, oval, or flattened masses, enveloped by the common cellular tissue. These masses, when submitted to a more minute state of division, are found to be composed of a number of small lobules, and these, in their turn, of an infinity of minute granules or particles, closely clustered together, which have been compared to the racimose arrangement of a bunch of grapes; the blood-vessels which lead to them forming a kind of pedicle, upon which the grains of fat are ingrafted." The adipose granules, according to the researches of some observers, would seem to be, as Dr. Geddings has described them, of an oval configuration: according to others, they are nearly spherical. M. Raspail states that these granulations exhibit various forms and dimensions, not only in different animals, but also in the same animal at different ages. In the ox and sheep they present distinct facets, so as to resemble the most regular crystals, reflecting the light in a manner similar to crystals of quartz. Those of the hog are rounded, oblong, turbinated, or kidney-shaped; while in insects they are of a turbinated form, in consequence of the large hilum with which they are furnished at the base.* The crystalline configuration of the adipose granules has been observed by Dr. Geddings himself in the ox and sheep; but he is inclined to regard it, with Weber, merely as an accidental condition resulting from their solidification after death.

Referring to the microscopical observations of M. Raspail upon

* Raspail, Nouveau Système de Chimie Organique. Dr. Henderson's Translation, p. 235.

the internal structure of the adipose tissue, Dr. Gedding subsequently observes,

"That each mass of solid adipose substance is surrounded by a strong membranous covering, or vesicle, in which no opening is perceptible. This mass is composed of an aggregate of smaller masses, likewise included within similar vesicles, of a thinner and more delicate character; and these secondary masses may be divided and subdivided until they are reduced to the primitive adipose granules, which are themselves contained in small delicate membranous vesicles, so attenuated as to be imperceptible to the natural eye, but which become manifest when they are prepared by immersion in boiling alcohol, and are in this state examined with the microscope. The adipose tissue, therefore, according to this view, seems to be composed of an external vesicle, from the inner surface of which others are formed of smaller size, and these latter are divided and subdivided, until they are reduced to those minute divisions which, being inflated with the adipose materials, form the primitive granules."

The close analogy of this structure with that of the cellular tissue of vegetables, must occur to the most superficial observer; the difference being chiefly, as is elsewhere remarked, in the character of the materials which are contained within the cells. But we cannot thence conclude, with M. Raspail and Dr. Geddings, that there is therefore no foundation for the opinions of William Hunter and Beclard respecting the existence of the adipose tissue as distinct from the cellular. Dr. Hunter's reasons in favour of this view, published in the second volume of the Medical Observations and Inquiries, have never been satisfactorily confuted; and Dr. Geddings scarcely does justice either to William Hunter or Beclard, in the imperfect summary which he gives of their statements.

When the cellular tissue is infiltrated with air, as in emphysema,—with serous fluid, as in anasarca,—with blood or pus, as in other states of disease,—or even artificially with oleaginous and other fluids,—the effused or introduced fluids readily pass from one part of this texture to another. This is especially the case with the serous infiltrations in anasarca, which, as is well known, spread throughout the body with extreme facility, always tending towards the parts in a depending position, as the scrotum and lower extremities, and readily yielding to the pressure of the finger. These phenomena, together with the evacuation of the loaded cellular tissue, by puncture or scarification, can scarcely be explained without supposing a free communication to exist between the cells or meshes of this texture. But, in the case of the natural adipose secretion, or of its excessive development, as in some morbid states, we do not find the oil or adeps passing freely throughout the body; neither do we observe it occupying in an especial manner the most depending parts, while those which are the most readily infiltrated by air, serum, or other fluids, as the eyelids and scrotum, are never, either in health or disease, the seat of an increase of the fatty secretions. We observe that Dr. Craigie, in Part I. of the Cyclopædia of Anatomy and Physiology, article *Adipose Tissue,* espouses the same views, and offers some additional reasons for considering this tissue as distinct from the cellular.

There are, it is true, good reasons for believing that not only do the increase and growth of the various modifications of cellular texture in vegetables result from the progressive development of what may be termed primary generating cells, but that also the vascular textures are in like manner derived from a similar origin; a vessel being in fact nothing more than an elongated cell open at both ends, or a number of cells arranged in a linear series, and communicating with each other at their point of contact so as to form a continuous tube. But similarity of origin proves neither identity of structure nor of function, and however strong may be the analogies between the general cellular texture of plants and the adipose tissue of animals, we cannot therefore regard the close vesicular texture of the latter as identical with or capable of performing the functions of the free, open, reticulated structure which constitutes the cellular tissue of man and animals.

The principal article connected with physiology contained in the parts which have been received by us is one on Absorption, by Dr. Jackson. There are some points connected with this subject upon which we were curious to ascertain the opinions held by our American brethren, and we expected that this article would convey the expression of their opinions with some authority. We regret however that the observations of Dr. Jackson are not such as we can regard with confidence. With respect to cutaneous absorption, one of the points upon which we were desirous of information, the view taken is in the affirmative, but the remarks upon this subject would have had more weight had they been extended and accompanied by a detail of the arguments and experiments by which the existence of the absorbing power in the true skin is proved. The observations upon alimentary absorption, that is, upon absorption as manifested in the gastro-intestinal mucous membrane, are equally hurried and unsatisfactory, affording but a very superficial view of this most important part of physiology; and here, as in the succeeding section upon absorption in the cellular tissue, many of the effects attributable to impressions upon the extremities of the nervous fibrils, as proved by the researches of Dr. Addison and Mr. Morgan, appear to be confounded with those arising from the actual absorption of deleterious agents. The second part of the paper, on the mechanism of absorption, is much more to the purpose, but we are inclined to think that some of the opinions advanced will startle by their novelty, if not by their depth, the cultivators of physical science on this side of the Atlantic. For instance, "Metaphysicians, by a *mental abstraction,* have made solidity and impenetrability properties of matter. This is, however, mere scholastic subtlety. So far as matter can be brought to our positive knowledge by the senses, porosity and penetrability are its constant properties. We have what may be regarded as almost the demonstration of the fact in the experiments of Graham and Dalton, but more especially in the very ingenious experiments of our intelligent collaborator, Dr. J. K. Mitchell, of this city, (Philadelphia), exhibiting the penetrativeness of the gases; and in the experiments of Dutrochet on the endosmose and exosmose of fluids through animal and vegetable tissues." "It may be inferred from these experiments," continues our author, "that whatever may be the properties of matter in its ultimate atoms, of which we know nothing, that *bodies*, whether inorganic or organic, are porous and penetrable by some other substances or matters." We cannot pause to argue with Dr. Jackson respecting the essential difference between bodies or material aggregates, and the molecules of matter of which these bodies consist, nor attempt to point out the many errors in the preceding passage, but we must be permitted to remark that such inaccuracies of expression as the following ought not to have escaped the revising pen of the editor. "Magendie instituted a series of experiments, the results of which led him not only to assert the existence of venous absorption, but he denied the same function to the lymphatics."

There is a very elaborate article on the Diseases of the Aorta, drawn up by Dr. Geddings, which requires more than a mere passing notice. Leaving the strictly anatomical details for consideration under the subject of arteries, he proceeds to describe the variations of this vessel from the healthy state under the heads of, 1. Inflammation of the Aorta. 2. Construction and Obliteration of the Aorta. 3. Dilatation. 4. Aneurism. 5. Wounds. 6. Ligature; and 7. Rupture of the Aorta. In the first section is a good description of the results of the inflammatory process as observed after death in the coats of the artery. The red, brown, or purplish stains, which are of not unfrequent occurrence in the lining membrane of the aorta and other large arteries, when unattended by other morbid changes, Dr. Geddings regards, with Laennec, Hodgson, Hope, and others, as arising from infiltration of the tissues after death. These appearances were at one time very generally looked upon as of inflammatory origin, and it may be doubted whether some recent pathological writers have not erred on the opposite side in ascribing too little importance to them as morbid phenomena. Thus, in an epidemic malady which prevailed amongst horses in the year 1825, reported by M. Andral, this peculiar redness was found to occur in the greater number of cases, and that even when the examination was made only half an hour after death, a period of time which seems almost too short to allow of the explanation contended for by these authors. The same remark applies to the redness of the lining membrane of the vascular system occasionally found in those who die of *Purpura hemorrhagica*, as the laxity of tissue and broken-down state of the blood are a part of the disease, and the effects of these changes upon any part of the animal fabric, although not necessarily to be regarded as indicative of a previous inflammatory state, are scarcely to be considered as an alteration depending merely upon the loss of vitality, more especially as they are found to occur in some instances during the life of the individual. The principal morbid changes in the aorta arising from inflammation, recognized by Dr. Geddings, are redness of its lining membrane, (which he characterizes as "less intense, less diffused, and not so abrupt in its termination in the neighbouring parts," as the redness proceeding from infiltration after death,) softening, induration, ulceration, effusion of plastic lymph, suppuration, atheromatous secretions, tumours within the coats, cartilaginous and osseous depositions, and lastly cancerous or encephaloid degenerations.

The following is the account given of the softening of the tissues.

"Softening of the coats of the artery is an early effect of inflammation. The lining membrane becomes soft and spongy, and loses its natural polished appearance. Portal* describes the case of a youth, who died in consequence of the repulsion of an exanthematous eruption, in whose body he found the internal membrane of the aorta red, tumid, and preternaturally soft. It can generally be detached from the fibrous tunic with great facility; and in some instances presents a slight villous appearance, similar to that exhibited by inflamed serous membranes. The fibrous coat becomes remarkably fragile, and is partially or completely divested of its elasticity. Softening likewise takes place to a limited extent in the cellular coat, which at the same time loses its natural pliability, but still remains somewhat resistent. It is, nevertheless, more yielding in this state than in its healthy condition; and this cause co-operating with the diminished cohesiveness of the other tunics, is doubtless instrumental in giving rise to the preternatural dilatations of the aorta which are so often observed. There is likewise great fragility of the coats of the aorta, when they are affected with chronic inflammation. Although more dense than in their natural state, they are so inordinately brittle, especially the middle coat, as to be easily lacerated."

We have been induced to make the foregoing extract, because it has been denied by Laennec and other competent observers, that softening of the coats of the arteries is really a characteristic mark of previous inflammatory action. Laennec states that the formation of a pseudo-membranous layer of plastic lymph, more or less adherent to the internal surface of the heart and blood-vessels, is the most incontestable sign of inflammation of that membrane, and with ulceration the only certain one:† and he even doubts whether red-

* Anat. Médicale, III. 127.

† Traité de l'Auscultation Médiat. Ed. 2. Vol. II. p. 607.

ness of the lining membrane of the arteries, though accompanied with swelling, thickening, puffiness, and an increased development of small vessels in the fibrous or middle coat, are to be received as proofs of inflammation in a subject considerably infiltrated, and the tissues of which are very humid.* Dr. Hope, quoting these passages,† refers to a case which he had recently met with, precisely such as Laennec describes, and which he, in conjunction with two other eminent medical men, had been inclined to consider as being inflammatory, until subsequent inspection proved the reverse. Notwithstanding these high authorities, however, we cannot hesitate to regard the changes described by Dr. Geddings as the result and evidence of a previously existing inflammatory state in the lining membrane of the larger blood-vessels, although we are quite willing to admit that such changes are not of very frequent occurrence. The fragility of the coats of the aorta described towards the conclusion of the passage quoted, as an effect of chronic inflammation, can scarcely be considered as a part of the softening process.

It is to be regretted that the enlightened author of this paper should have suffered himself to be trammelled by the usage of an exclusive and erroneous pathology, and thus have classed, under this head of Inflammation, such diverse morbid products as tumours, cartilaginous and osseous depositions, and cancerous degenerations. The reason adduced for this proceeding, that, in the present state of our pathological knowledge, the question of the origin of such variations from the healthy state cannot be decided, and therefore that it is better to group them all under one head, than to make a multiplicity of divisions,—is anything but conclusive. In the present imperfect state of our knowledge, there must always remain a vast number of effects which we are unable to refer to their causes; but, although we may not have it in our power to point out the true origin of any specific deviation from the healthy condition, the deficiency is surely no reason for adopting an acknowledged error. It is time that this method of glossing over ignorance with a name should be abandoned, and that our systems, though perhaps of necessity imperfect in their construction, should no longer be avowedly false. The morbid changes alluded to are undoubtedly to be ascribed to an altered state of the nutrition of the organs in which they are found to occur, and may be simply classed as such until the progress of our knowledge shall enable us to give a more satisfactory account of them.

The symptoms of inflamed aorta are very obscure, and the diagnosis of this affection consequently uncertain. Dr. Geddings quotes the summary given by Frank in his *Praxeos Medicæ Universæ Præcepta;* but it contains nothing satisfactory upon the subject. There is no one symptom or assemblage of symptoms

* Ibid.

† Cyclopædia of Practical Medicine. Article, *Arteritis;* Vol. I. p. 144.

which can be fixed on as characteristic, and in every instance the physician must be indebted rather to his own sagacity and tact for the discovery of this obscure morbid condition, than to any prescribed rules of diagnosis. The same observations necessarily apply to the treatment of such cases; though, when inflammation is suspected to exist in the aorta or larger blood-vessels, it is to be treated upon general principles, modified in their application according to the circumstances of the individual case.

The succeeding section, upon Constriction of the Aorta, contains much curious information, and is well worthy of an attentive perusal.

The subjects of Dilatation and Aneurism are treated at considerable length in the third and fourth sections. Dilatation of the Aorta is thus defined:

"By this term is meant a general dilatation of all the coats of the aorta, occupying more or less the extent of that vessel. It is distinguished from aneurismal dilatation, by the latter being generally, though not always, confined to a limited extent of the aorta; by the presence in aneurism of a tumour more or less manifest, either occupying the entire circumference of the vessel or confined to a portion of its walls; and, finally, by the absence, in simple dilatation, of those laminæ of coagulated blood and fibrinous depositions, which always form within an aneurism, and become intimately connected with the inner surface of the sac."

The distinction here attempted to be established between simple dilatation and true aneurism is, in many instances, more of a nominal character than one founded upon correct observation. The extent of the change does not, as Dr. Geddings has himself remarked, in all instances afford a sufficient criterion; and fibrinous depositions within the dilated vessel, especially in cases when its lining membrane is not in a healthy state, have been found when the dilatation was simple, and not sacculated or aneurismal. Examples of this kind, related by Messrs. Bertin and Bouillaud, and by Mr. Guthrie, are indeed referred to by Dr. Geddings; and, with respect to the second mark of distinction, it is subsequently observed, that "the tunics are often distended only in one direction." We would not, however, be understood to follow Scarpa in denying the existence of what has been termed true aneurism, as distinct from simple dilatation of the artery, but merely to state that the line of demarcation between these morbid conditions is not always distinctly to be made out, and certainly not with that precision which some pathologists contend for.

Under the head of Aneurism of the Aorta, we find treated of, in succession, the anatomical characters of true aneurism, of false aneurism, and of mixed aneurism, the influence exercised by aneurism of the aorta upon the adjacent parts; the causes, symptoms, and diagnosis; and, lastly, the spontaneous cure and medical treatment. The observations upon each of these subjects are well worthy of attentive perusal, and especially those on the influence upon adjacent parts, and on the symptoms and diagnosis. The obscurity in which cases of aneurism of the aorta are not unfrequently involved, and in particular those instances where the thoracic portion of this vessel is the seat of the dilatation, is a source of disappointment and regret to the intelligent and well-informed practitioner: of disappointment, because he will, if in extensive practice, recollect but too many instances where his efforts have been foiled from being directed against symptoms presumed to arise from a cause more within the reach and resources of art; and of regret, because he will know that, although a cure could not have been effected, much suffering might have been spared in the unavailing employment of active and painful remedies.

Upon this subject, however, we cannot now enter, but must be content with referring to Dr. Geddings's paper, to Dr. Hope's Treatise on Diseases of the Heart, and especially to the article *Aneurism of the Aorta*, in the Cyclopædia of Practical Medicine, by the same writer, in which the physical signs of this lesion are set forth with as much precision as the subject at present admits of.

The following remarks upon the treatment of aneurism of the aorta, although somewhat at variance with received opinions, are deserving of attentive consideration. After quoting the mode of treatment by free depletion, recommended by Dr. Hope in the treatise before referred to, Dr. Geddings proceeds:

"While we are willing that such a course should be adopted as a general rule, especially in those whose constitutional powers have not been seriously impaired, we feel assured that, in a large proportion of cases, blood must be more sparingly drawn, if we would not jeopard the life of the patient. There is, indeed, abundance of evidence to prove that too much depletion, and too severe a regimen, will often thwart the fulfilment of the very objects we have in view. The bad effects may, as previously remarked, either consist in the impairment of the plastic or cohesive powers of the blood, by depriving it of its fibrine; in the development of that peculiar preternatural throbbing of the vascular system, which is so wont to supervene upon copious abstractions of blood; and, finally, an enfeebling of the cohesiveness of the coats of the artery, rendering them more friable, and consequently more prone to rupture under the distending influence of the blood. The truth of the last inference has been fully confirmed by the extensive experience of Dupuytren, who has repeatedly remarked, that internal aneurisms, treated by Valsalva's method, are apt to increase more rapidly in size, and finally rupture; consequences which he explains upon the supposition that the depletion weakens the coats of the arteries more than it does the action of the heart."

Dr. Copland, in his Dictionary of Practical Medicine, gives the same opinion as to the effects of large and frequently repeated abstractions of blood. "I believe," says he, "that there is no position in pathology more firmly established, since it was insisted on by John Hunter, than that whatever greatly lowers the vital energies will impede the formation of coagulable lymph and fibrinous coagula, especially in diseased vessels; and that increased rapidity of the circulation, throbbing of the arteries, abstraction of the fibrine and red globules of the blood, by repeated or large depletions, and the absorption of serous, watery, or unassimilated materials into the current of the circulation, in order to supply the place of the portion of the blood abstracted, will, with other effects, inevitably tend to prevent those changes from taking place which we wish to bring about." In these observations we fully concur; and we feel assured, from some experience in these cases, that, after the plethoric condition of the constitution has been subdued, an occasional small bloodletting, with moderate diet, perfect quietude both of body and mind, and the employment of sedatives, will form the most efficient and satisfactory mode of treatment.

It will be fresh in the recollection of many of our readers, that, in the winter of the year 1832, a young girl died suddenly at the Hôtel Dieu, Paris, while undergoing the removal of a tumour from the neck, by the late Baron Dupuytren, and that the fatal event was attributed to the admission of air into the vein. A similar accident had previously occurred, in the year 1818, to M. Beauchêne, in removing a tumour from the neck of a young man, at the Hôpital St. Antoine. Since then it has been observed upon other occasions in the practice of several distinguished surgeons. As recent examples, we extract from the article *Air* the following cases, which occurred to Dr. Warren, by whom they are related.

A man, aged sixty, was admitted into the Massachusetts General Hospital, on the 16th October, 1830, with an ulcerated cancerous affection of the left side of the face and neck, involving the parotid, submaxillary and sublingual glands, and all the textures excepting the bone. The patient being desirous of having an operation performed for its removal, it was thought right, in the first instance, to secure the carotid artery.

"An incision for this purpose was begun opposite the thyroid cartilage, and carried two inches downwards. The platysma muscle was divided; the edge of the mastoid exposed, and dissected. Thus far only a few drops of blood were discharged. The face of the sheath of the great vessels was a little uncovered, when a small effusion of venous blood appeared under the knife, and checked the operation. At that instant a very distinct sound was heard, like the passage of air through water. A few bubbles were seen in the venous blood, the flow of which was immediately arrested by applying a finger on the part. The patient exclaimed 'I am faint!' On regarding his countenance, it was not pale, but livid, almost black, and the muscles agitated by a convulsive motion. The respiration became deep, laboured, and stertorous, like that of apoplexy. The pulse being examined at the wrist, was found distinct, but very slow. The wound not bleeding, and very little blood having been lost, the temporal artery was opened, and the blood flowed from it with great freedom. As it flowed, the respiration became more frequent and less laborious, the pulse at the wrist more natural. The leaden colour in the cheeks assumed a reddish tinge, and the alarming character of the symptoms was evidently diminished. About twenty minutes elapsed during these changes. At the end of half an hour it was thought safe to remove the patient to his bed, where he lay in a state of insensibility for two hours; at the expiration of which he awaked as from sleep, still breathing like an apoplectic. The night was passed without any accident; and on the following morning he was as well as usual, with the exception of a moderate soreness over the thorax, and a headach."

The operation was performed seven days afterwards, without tying the carotid artery; and the man recovered without experiencing any bad symptom.

A married woman, aged thirty-three, had a hard moveable tumour of the right breast, involving the whole gland; the glands in the axilla being also diseased. An operation was performed on the 24th December, 1831. The patient having been placed in a proper position, and the breast partially dissected out, the axillary glands were then cautiously detached from the great axillary vessels to which they adhered. The separation was nearly effected, when a vein at the outer part of the axilla was divided, and a small quantity of venous blood discharged.

"Scarcely was this done, when the patient struggled, her complexion changed to a livid colour, and at the same instant the bubbling or clucking noise, which had not been noticed before, was heard, though indistinctly; but the place from which it issued was not visible, the surrounding skin and fat lying over it. On this, the axilla was immediately compressed. The patient became insensible, breathing as in apoplexy. The tumour was at once separated. The posture of the patient was changed, and she was supported by those around. Some brandy was poured down the throat, and ammonia introduced into the nostrils. The pulse, however, became less distinct every instant. Cloths dipped in hot water were thrown over the extremities. Strong frictions were applied to the chest, and to all parts of the body. Considerable quantities of brandy were again poured down the throat. At this moment, the livid colour of the cheeks gave place to a suffusion of vermilion red; and no glow in the cheek of a youthful beauty ever gave one so much pleasure as that flush. But the flush soon passed off: the lividness reappeared, the respiration became more feeble, pulse at the wrist scarcely perceptible; and, notwithstanding the redoubled applications of external heat and moisture, the extremities and the whole body cooled rapidly, and presently the respiration ceased."

The larynx was opened as a last effort, and the lungs regularly inflated for about twenty minutes, but without effect. No inspection of the body was obtained.

We regret being compelled to pass over an excellent article upon the anatomy, physiology, and pathology of the Anus, by Dr. Reynell Coates; and a most elaborate account of Antimony, its

Chemical History, Pharmaceutical Preparations, Therapeutical Applications, and Toxicology, by Dr. Franklin Bache. Some other medicinal agents from the mineral kingdom are also illustrated by Dr. Bache; while those from the vegetable kingdom are written by Dr. R. E. Griffith and Dr. Wood. These last are in general carefully written, and present some features of considerable interest: they are especially worthy of attention, as giving an account of many native remedies employed in the practice of the American physicians.

The articles, generally, partake more of the character of compilations from other authors, than of original essays; but many of them evince much research, and considerable judgment in the selection of the materials from which they have been compiled. Upon the whole, the work is highly creditable to the industry, professional acquirements, and judgment of the physicians engaged in it, and promises to be of considerable utility as a digest of medical literature. There is one great defect, however, existing in the arrangements for publication, which we feel ourselves called upon to notice: we allude to the slowness of its progress. The first Part bears the date of July 1833, and contains the following notice on the cover: "The work will be published in parts, averaging 112 pages each. It is expected that it will be completed in forty parts, making eight large volumes." The seventh part, which only proceeds as far as Apoplexy, appeared in April 1835. At this rate of proceeding, the work, at the same time that it will much exceed its contemplated limits, can scarcely be completed in less than thirty years, and may possibly require a much longer period.

The mode in which it is got up, although not in the best style of the American press, is very creditable to the publishers. It is, indeed, gratifying to observe the degree of perfection to which this wonderful people have already arrived in nearly all the departments of the arts; and it is particularly delightful to us to reflect that the greatest and most divine of all arts, the art of healing, flourishes throughout the vast extent of that great country, with a degree of vigour, soundness, and activity, which reflects infinite credit on the members of the profession generally, and adds fresh glories to the common language and common mother-land of all who think and write in the English tongue.

